# Comparing body image dissatisfaction between pregnant women and non-pregnant women: a systematic review and meta-analysis

**DOI:** 10.1186/s12884-023-05930-w

**Published:** 2023-10-04

**Authors:** Anna Elizabeth Crossland, Lydia Munns, Elizabeth Kirk, Catherine Elizabeth Jane Preston

**Affiliations:** 1 Department of Psychology, University of York, Heslington, YO10 5DD UK; 2 School of Psychology and Sport Science, Anglia Ruskin University, Cambridge, CB1 1PT UK

**Keywords:** Body image dissatisfaction, Pregnancy, Meta-analysis, Systematic review, Physiological changes

## Abstract

Body image dissatisfaction refers to negative thoughts and feelings individuals have towards their own body appearance and this is thought to be affected by the physiological changes that occur during pregnancy. There are two main conflicting theories as to the effect pregnancy has on body image dissatisfaction: 1) Pregnancy related changes are in direct conflict with social ideas of female beauty (e.g. weight gain) and so increase body image dissatisfaction; 2) Due to changes in expectations of bodily appearance during pregnancy, women are liberated from social ideals at this time and thus body image dissatisfaction would decrease. This study aimed to assess these theories by synthesising the current literature. Six databases were searched, and 2,017 study abstracts were screened based on strict inclusion and exclusion criteria. Following screening and quality assessment by two blind reviewers, 17 studies (comprising 17 effect sizes) were subject to full review and meta-analysis following PRISMA guidelines. These studies included cross-sectional, longitudinal and retrospective designs. Results varied with some studies showing women to feel more positive about their body during pregnancy, others showing a more negative body experience and yet others showing no statistical difference. Overall the analysis showed no statistical difference in body image dissatisfaction between pregnant women and non-pregnant women (*p* = 0.39). Any changes that do occur are heterogeneous and likely to be largely dependent on the individual experience as well as moderator variables and other factors such as differences in methodology of research studies. Studies in this field of research would benefit from more explicit and complete reporting of data and key variables, in order to allow early intervention for women who display body image dissatisfaction in pregnancy.

**Supplementary Information:**

The online version contains supplementary material available at 10.1186/s12884-023-05930-w.

## Background

Women can be subject to cultural pressures and expectations about how their body looks [1], which can lead to body image dissatisfaction (negative subjective evaluations of one's physical body, such as figure, weight, stomach and hips; [2]). During pregnancy women experience vast and very noticeable physiological changes, such as the abdominal area growing, overall weight gain [3], changes in posture and gait [4], and changes in the appearance of the hair and skin [5]. These changes are not only in direct conflict with Western socially constructed ideals of female body appearance, such as having “a flat stomach, thin waist, boyish hips, long legs, well-developed breasts, well-defined muscles, and flawless skin” ([6]; p11), but also ideals of female appearance in other areas of the world such as the Middle East [7] and Asia [8, 9], where promotion of a slim female physique is also widespread. This can cause females to develop body image dissatisfaction due to internalisation of societal expectations about appearance [10].

Qualitative research supporting the idea that women’s satisfaction with their body image will worsen during pregnancy demonstrates that an increase in weight during pregnancy can cause pregnant women to ‘feel fat’, particularly in areas aside from their abdomen such as their face and limbs [11] and especially during early pregnancy when they do not look obviously pregnant, but their abdomen is growing in size [12]. Other women describe the feeling of their body being out of their control, because they are aware that their body will change but they cannot stop it happening or control how it changes, which can cause them distress and to perceive their body negatively [11]. Images in the media depicting unrealistic pregnant women, often edited to remove uneven skin tone and stretch marks can lead to increases in body image dissatisfaction amongst pregnant women, suggesting that unrealistic expectations of how women ‘should’ look during pregnancy can have a negative impact on how women feel about themselves [13].

However other women report having a more positive bodily experience during pregnancy than when not pregnant [14]. This could be as they no longer compare their own body to the thin ideal body type, which according to Thompson et al. [15] is a pivotal part of social influence on body image dissatisfaction. This explanation suggests that pregnant women accept that their bodily changes are out of their control temporarily, relieving the pressure to try to conform to thin ideals [16]. This could be due to changing expectations of how the body will look as pregnancy progresses, or due to the awareness of how transient weight gain could be [17]. Some women report that the improvement in body image satisfaction during pregnancy is because of the adjusted functionality of the body [18], meaning less focus on how the body looks and more on its functionality [19] possibly because weight gain has a clear function such as the increasing weight of the fetus and amniotic fluid, or fat stored for breastfeeding causing increased breast size and weight. Body image may also become less of a priority during pregnancy [20] as focus turns to the fetus’ health and the new maternal role, focussing more on ideals of motherhood and therefore judging their worth by different criteria [19]. Other women report improved body image satisfaction because of positive feedback on their pregnant body shape from others, receiving compliments on their ‘blooming’ appearance [18]. This is further illustrated by women reporting adjustments in their ideal body size over the course of pregnancy [12], suggesting that women feel liberated from societal body ideals [18] and reflecting realistic ideals. It also indicates acceptance of their weight and size increasing, acknowledging that changes in diet and weight gain are ‘allowed’ during pregnancy [21].

The apparent incongruence in previous literature could be due to several factors related to the individuals included in any particular study, such as gravidity (the number of times a woman has been pregnant), age, pre-pregnancy BMI (body mass index), gestational weight-gain and mental health status. For example, some evidence suggests that primigravidae (women in their first pregnancy) have more positive attitudes towards their body compared to multigravidae (those who have been pregnant before), and report feeling more attractive than multigravidae [22]. However, multigravidae samples, by their nature, tend to be older and it is thought that feelings towards the body [23] and body appreciation [24] improve with age. A further potential influencing factor for body image dissatisfaction and differences related to gravidity is overall body size; weight gain from previous pregnancies can influence the pre-pregnant self to which pregnant women may be comparing themselves [20]. Indeed, multigravidae samples are found to have a higher average BMI compared to primigravidae [25] and higher BMI is associated with greater body image dissatisfaction, particularly amongst females [26], as well as being linked to maternal well-being and eating behaviour during pregnancy [27]. Furthermore, women who gain more than the recommended amount of weight during pregnancy have reported more negative body image, particularly later in gestation [28]. Correlations have also been reported between pregnancy body image dissatisfaction and perinatal depression [29], postpartum depression [30], antenatal anxiety [31] and long-term anxiety [32], which suggests that mental health status could be related to body image dissatisfaction at this time.

These potential contributing factors illustrate the importance of considering differences concerning the individual that could affect pregnancy body image dissatisfaction. However, there are also factors related to the study methodology that may also be important, for example the time period of pregnancy when the study is conducted and the study design used. As the fetus grows, different parts of the pregnant mother’s body grow, such as breasts, hips, thighs and abdomen. Thus, because of these continual bodily changes, perceptions and feelings towards the pregnant body may also be changing throughout the course of pregnancy [12, 33]. This may mean that the stage of pregnancy at which women are asked about their feelings towards their body could impact the level of body image dissatisfaction reported [34–36]. Furthermore, different studies capture body image dissatisfaction in pregnant and non-pregnant samples using different methods, such as cross-sectional between designs and longitudinal or retrospective within designs. These factors could influence the apparent outcome of the studies.

It is important to understand how pregnancy may impact body image dissatisfaction because this may have implications on a woman’s mental health [37], and as pregnancy is a period of a woman’s life in which there is enhanced risk for mental illness [38], research suggests that body image dissatisfaction can moderate this [39]. Many women who display body image dissatisfaction during pregnancy also exhibit depression and anxiety both postnatally [21, 31] and longer-term [32]. This can lead to negative emotional, cognitive and behavioural outcomes for the child [40], as well as poor quality mother-infant interactions [41]. Body image dissatisfaction in pregnancy is also associated with weaker bonds with the fetus before birth (antenatal attachment; [42]) as well as with a reduced intention to breastfeed and actual breastfeeding duration [43]. In addition, body image dissatisfaction has been linked with physical illness as the expectant mother may engage in detrimental practices such as unhealthy eating, dieting, purging and fasting [44]. This can have unwanted negative effects on the fetus such as low birth weight and premature birth [45]. Conversely positive body image is thought to protect against negative mental and physical health [46], including lower reporting of symptoms of depression [47], improved self-esteem [46] and engaging in more positive health related behaviours such as reduced alcohol and tobacco intake, behaviours protecting against cancer such as sunscreen use, seeking routine medical attention and preventative sexual health behaviours [48].

Due to the important role of body dissatisfaction in pregnancy for the expectant mother and fetus it is therefore important to understand how body image dissatisfaction may change when pregnant compared with when not pregnant. There are many reviews and meta-analyses that consider postpartum body image (dis)satisfaction (e.g. [11, 20, 30]) and systematic reviews during pregnancy (e.g. [49]) but to the authors’ knowledge there is currently no systematic review and meta-analysis of studies investigating differences in body image dissatisfaction between pregnant and not pregnant samples. It is important to synthesise the literature to allow for an overall view due to inconsistencies in current research; with some studies supporting that body image improves due to a release from social ideals and/or emphasis on function over appearance whilst other studies suggest that body image worsens during pregnancy because of a deviation from social ideals when pregnant. The current study therefore aims to review and synthesise articles with quantitative measures of body image dissatisfaction, using independent measures designs (cross-sectional) and repeated measures designs (longitudinal and retrospective), to understand whether and how body image dissatisfaction changes in pregnant women compared to non-pregnant women. It also aims to analyse the role that various moderators play in body image dissatisfaction in pregnancy. To assess whether body image dissatisfaction changes when pregnant, a random effects meta-analysis will be used to statistically synthesise research from pregnant and non-pregnant women that fits inclusion criteria for analysis.

## Method

### Systematic literature review

A detailed protocol was developed and registered on prospero [CRD42021288692], comprising eligibility, inclusion and exclusion criteria, which were decided a priori. A comprehensive literature review was undertaken from several online databases from commencement to March 2022: Scopus, Psychinfo, Web of science, Pubmed, Cochrane library and Embase, in accordance with the Preferred Reporting Items for Systematic Reviews and Meta-Analyses (PRISMA) guidelines [50]. Unpublished studies and those reported in the grey literature were also sought. Search terms of “pregnancy” and “body image” and their synonyms were used (Table 1), which had been gleaned from a scoping literature review of other relevant systematic reviews (e.g. [11, 30, 51]) of body image in pregnancy.

**Table 1:** Synonyms used for search

Terms for pregnancy	Terms for body image
Pregnan*	Body image
Prenatal	Body satisfaction
Antenatal	Body dissatisfaction
*gravid*	Body concern
*parous*	Body preoccupation
Gestation*	Body attitude
Perinatal	Body image disturbance
	Body image distortion

NOT case study, animal

* denotes truncation

Studies that fit strict criteria were eligible for inclusion. The studies had to include quantitative, validated self-report measures of women’s body image dissatisfaction during pregnancy and from a non-pregnant sample, from which an effect size could be calculated. The studies could include any appropriate design, including repeated measures, whereby one participant group were used, where each participant was either pregnant at the time of the study and reflecting back on their pre-pregnancy body image dissatisfaction, or longitudinal whereby women were questioned repeatedly from before they were pregnant to during pregnancy; and also cross-sectional studies (independent measures), whereby participants were either pregnant or not pregnant at the time of the study and so used two independent samples. Only English language studies were included due to English being the primary language spoken by the researchers. Articles could be from any publication year, as long as they were available through the search engines listed. Studies of women having single or multiple pregnancies were included, as were studies of women in all relationship status’ and of all age groups. “Pregnant” was classed as any woman who self-reported or was medically acknowledged as being in the gestational period. “Non-pregnant” was classed as not pregnant at the time of the study in the case of concurrent studies, or using a retrospective measure asking pregnant women to recall before they became pregnant, or longitudinal whereby women were questioned before becoming pregnant then again during pregnancy.

Strict exclusion criteria were also applied: Studies measuring body image dissatisfaction soon after pregnancy (in the postnatal stage) as the only non-pregnant time point were excluded, as literature suggests that body image satisfaction differs in the postnatal period [52]. Only studies measuring body image dissatisfaction before pregnancy or between pregnancies, as well as measuring body dissatisfaction during pregnancy were included. Other specific a priori exclusion criteria were also applied in terms of the study type and sample. Studies gathering qualitative data or using case studies were excluded, as were reviews, although the authors checked the reference section of relevant reviews to identify any studies to be included in the screening stages (e.g. [11, 30, 51]). Studies that tested women with clinical diagnoses of eating disorders or studies using non-human samples were also excluded, as were studies published in non-English language.

### Screening process

**Fig. 1:**
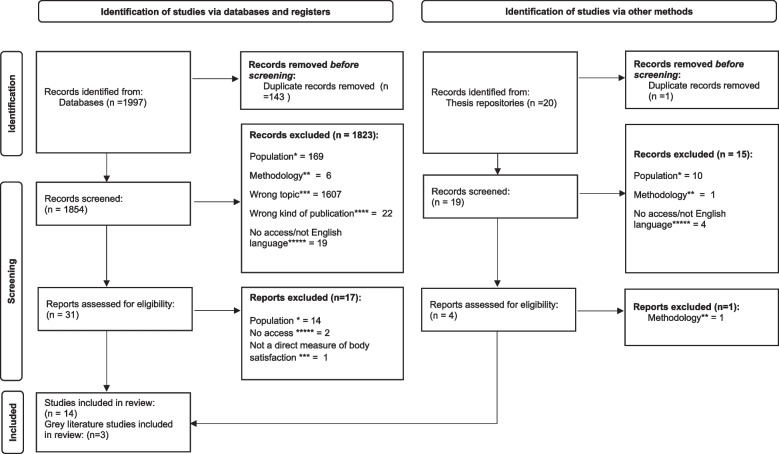
The study selection process in a PRISMA diagram *Absence of non-pregnant comparison, non-pregnant comparison was postpartum, no measure of pregnancy BID, clinical sample. **Qualitative data, pilot study, experimental research. ***Measured variables like self-esteem, confidence. ****Book, theoretical description. *****Article not available online, including through institutional availability and despite contacting authors

The initial search yielded 2,017 studies. Screening was conducted following the PRISMA guidelines [53], using Rayyan [54], which is an online software tool for conducting, organising and sharing systematic reviews. Rayyan was chosen due to its flexibility and ease of use, as well as the ability to be blind to the other reviewers’ coding and to add labels for excluded papers. Grey literature was searched, and authors were contacted for unpublished data. Two reviewers (AC and CP) screened all non-duplicate studies independently against the eligibility criteria using information from the title and abstract. Each article was marked independently by each reviewer with a “yes”, “no”, or “maybe”, and articles marked “no” by both reviewers were excluded from the analysis. Articles were removed for many reasons, but notably most exclusions at this stage were because the title and/or abstract were clearly not related to the topic of body image dissatisfaction in pregnancy. Following title and abstract screening, the full text of any articles marked ‘yes’ or ‘maybe’ were read independently by the same two reviewers to determine whether they met the inclusion criteria. After this stage 89 papers remained for full text review. Further articles were excluded at this stage for many reasons such as sample (for example clinical samples which were excluded due to having extreme body image dissatisfaction which is qualitatively different to non-clinical samples, and being a minority population), not measuring body image dissatisfaction or not including a non-pregnant comparison, as per the exclusion criteria (see Fig. 1). Agreement was very high (99.1%), and any disagreements were discussed by the reviewers (0.9%); full agreement was reached after discussion and closer inspection of the papers, so it was not necessary to involve a third reviewer at either stage. Thirty-five papers were fully assessed for eligibility, of which 17 studies met all inclusion criteria [12, 14, 31, 36, 55–67]; Table 3). This process is shown in the PRISMA diagram, Fig. 1.

### Data extraction

Studies that fitted the criteria were subjected to a methodological quality assessment to determine the extent to which a study may be subject to threats to validity, as recommended by The Joanna Briggs Institute (JBI; [68]). The JBI Critical Appraisal Checklist has been recommended for an analytical cross-sectional study [69], including questions about the appropriateness and clarity of the measures and sample, as well as identifying and dealing with confounding factors. Each of the eight questions were rated high quality, low quality, unclear or not applicable (see Table 2), with a random sample checked by all authors, with high agreement. Studies were rated as high if 5 or 6 categories out of 6 were classed as high quality, and moderate if 3 or 4 categories were classed as high or moderate quality (see Table 3).

**Table 2:** JBI Quality assessment

	**Were the criteria for inclusion in the sample clearly defined?**	**Were the study subjects and the setting described in detail?**	**Was the exposure measured in a valid and reliable way?**	**Were objective, standard criteria used for measurement of the condition?**	**Were confounding factors identified?**	**Were strategies to deal with confounding factors stated?**	**Were the outcomes measured in a valid and reliable way?**	**Was appropriate statistical analysis used?**
Chan et al. (2020) [31]	High	High	N/A	N/A	High	High	High	High
Clark and Ogden (1999) [60]	High	High	N/A	N/A	Unclear	Unclear	High	High
Crossland et al. (2022) [55]	High	Unclear	N/A	N/A	High	High	High	High
Davies and Wardle (1994) [57]	Low	High	N/A	N/A	High	High	High	High
Duncombe et al. (2008) [56]	Unclear	High	N/A	N/A	Low	Low	High	High
Fuller-Tyszkiewicz et al. (2020) [65]	Low	High	N/A	N/A	Unclear	Low	Unclear	High
Gough (1998)* [64]	High	High	N/A	N/A	High	Low	High	Unclear
Harrison et al. (2019) [63]	High	High	N/A	N/A	High	Low	High	Unclear
Inanir et al. (2015) [36]	High	High	N/A	N/A	Unclear	Low	High	Unclear
Lombardo (2001)* [61]	Unclear	High	N/A	N/A	Unclear	Low	High	High
Loth et al. (2011) [14]	Unclear	Low	N/A	N/A	High	High	High	High
McCarthy (1998)* [70]	High	High	N/A	N/A	High	Low	High	Unclear
Meireles et al. (2021) [66] and Hudson et al. (2021)** [67]	Unclear	Unclear	N/A	N/A	Unclear	N/A	High	N/A
Pascoal et al. (2019) [62]	Low	Low	N/A	N/A	Unclear	High	High	Unclear
Pieta et al. (2021) [58]	High	Unclear	N/A	N/A	Unclear	Low	High	High
Ruggieri et al. (1979) [59]	Unclear	Unclear	N/A	N/A	Low	Low	High	High
Skouteris et al. (2005) [12]	Unclear	High	N/A	N/A	High	Unclear	High	High

* denotes the studies retrieved from grey literature

**Meireles et al. (2021) [66] and Hudson et al. (2021) [67] is formed from two studies provided after contacting the authors, one using pregnant participants and the other using non-pregnant participants, conducted in the same laboratory using the same measurement, and therefore is considered comparable as one study

**Table 3:** Summary of body (dis)satisfaction measures used in each study (*K* = 17) of the meta-analysis, and time points at which data was collected/used. In cases of multiple results, justification of which were chosen and why

*Authors*	*Quality rating*	*Measures taken *	*Measure used for analysis*	*Justification*	*Data collection point*
Chan et al. (2020) [31]	High	Body dissatisfaction (BD) subscale of EDI (Eating Disorder Inventory; [71]; 4 questions developed for this study	Body Dissatisfaction subscale from EDI	Validated measure, including measuring discontentment with the overall shape and size of 10 body regions. Drive for thinness may not apply during pregnancy as pregnancy contravenes thin ideals	Retrospective measure of 6 months pre-pregnancy (T0; collected at T1), Trimester 1 (T1)*, Trimester 2 (T2), Trimester 3 (T3)*, Postnatally (6 weeks postpartum)* (T4)
Clark and Ogden (1999) [60]	High	Restrained eating subscale of Dutch Eating Behaviour Questionnaire [72]; 8 item version of Body Shape Questionnaire [73]	Body Shape questionnaire	Measures concerned with body image dissatisfaction – more relevant than restrained eating behaviours	Retrospective measure of 3 months pre-pregnancy, Trimester 2, Non-pregnant control asked their average body image over last 7 months
Crossland et al. (2022) [55]	High	Body Cathexis scale [74]; Body Understanding Measure in Pregnancy Scale (BUMPS; [42]	Body Cathexis scale	BUMPS is only relevant to pregnant women and therefore does not provide a comparison for non-pregnant women	All trimesters mixed to form the pregnant sample (8% in trimester 1, 38% in trimester 2, and 54% in trimester 3), Non-pregnant control
Davies and Wardle (1994) [57]	High	Drive for thinness (DT) subscale and Body dissatisfaction (BD) subscale of EDI [71]; Dutch Eating Behaviour Questionnaire [72]; Other scales developed for the study	Body Dissatisfaction subscale from EDI	Validated measure, including measuring discontentment with the overall shape and size of 10 body regions. Drive for thinness may not be relevant during pregnancy as pregnancy contravenes thin ideals	33.4 weeks gestation, Non-pregnant control
Duncombe et al. (2008) [56]	Moderate	4 subscales from Body Attitudes Questionnaire (BAQ; [75]; Contour Drawing Rating scale [76]; Pregnancy figure rating scale ([12]; Dutch Eating Behaviour Questionnaire [72]	BAQ; feeling fat subscale	Contour Drawing Rating scale is images of non-pregnant women so is not relevant as a comparison for pregnant women; likewise Pregnancy figure rating scale is only relevant to pregnancy, not control and is therefore not comparable with the other studies. BAQ is more relevant to pregnancy, for example because the focus is on body satisfaction rather than eating behaviour	Retrospective measure of 3 months pre-pregnancy tested at T1, 18.5 weeks (T1), 26.6 weeks (T2)* 34.5 weeks (T3)*
Fuller-Tyszkiewicz et al. (2020) [65]	Moderate	Body Image in Pregnancy Scale (BIPS; [77]; Body Attitudes questionnaire (BAQ; [75]	BIPS: Appearance subscale – overall appearance score from average of 21 appearance ratings, edited for non-pregnant participants to exclude the words ‘ during pregnancy’ from each scale	More relevant to body image foci in pregnancy	Trimester 1, Trimester 2, Trimester 3, Non-pregnant control
Gough (1988) [64]	High	Drive for thinness (DT) subscale and Body dissatisfaction (BD) subscale of EDI [71]	Body Dissatisfaction subscale from EDI	Validated measure, including measuring discontentment with the overall shape and size of 10 body regions. Drive for thinness may not be relevant during pregnancy as pregnancy contravenes thin ideals	Retrospective measure of 12 months pre-pregnancy, 18–22 weeks Follow up at 34 weeks*
Harrison et al. (2019) [63]	High	Body Esteem scale for adolescents and adults [78]; EAT-26 Eating attitudes test ([79]	Body Esteem scale for adolescents and adults; general feelings about appearance subscale	Eating attitudes test is less relevant during pregnancy as eating attitudes and behaviours may be in response to body signals (such as nausea or cravings) rather than body attitudes. BESAA includes weight satisfaction, esteem from body and importance of opinions of others, which are all more applicable to pregnancy	22 weeks (reported in the qualitative analysis part of the paper), Non-pregnant control
Inanir et al. (2015) [36]	High	Body Cathexis Scale (BCS; [74]; Rosenburg Self-esteem scale [80]	BCS	BCS measures strength and direction of feeling towards various body parts so closely links with body image dissatisfaction. The meta-analysis does not intend to measure self-esteem; this is a qualitatively different concept	Trimester 1, Trimester 2, Trimester 3, Non-pregnant control
Lombardo (2001) [61]	High	Multidimensional Body Relations Questionnaire [81]	Appearance evaluation subscale	Most similar to the other rating scales	All trimesters mixed to form the pregnant sample, Non-pregnant control
Loth et al. (2011) [14]	High	Body Shape Satisfaction Scale [82]	Body Shape Satisfaction Scale	Only one scale was used in this study	Stage of pregnancy not stated, Non-pregnant control
McCarthy (1998) [70]	High	Multidimensional Body Relations Questionnaire [81]	Appearance evaluation subscale	Most similar to the other rating scales	Trimester 1 (M = 10.5 weeks pregnant)*, Trimester 3 (M = 32 weeks pregnant), Non-pregnant control
Meireles et al. (2021) [66] and Hudson et al. (2021) [67]	High	Body Appreciation Scale [83]); Rosenburg Self-esteem scale [80]; Eating Attitudes Test-26 [79]	Body Appreciation Scale	The Eating Disorder symptoms scales are not as relevant as the Body Appreciation Scale as this focuses on satisfaction with the body	Trimester 1, Trimester 2, Trimester 3, Non-pregnant control
Pascoal et al. (2019) [62]	Moderate	Global Body Dissatisfaction Scale (GBDS) – subscale of Body Attitudes Test [84]	Global Body Dissatisfaction Scale (GBDS)	Only one scale was used in this study	All trimesters mixed to form the pregnant sample (51.3% in trimester 2, 23.1% in trimester 1 and 25.6% in trimester 3), Non-pregnant control
Pieta et al. (2021) [58]	High	Multidimensional Body Relations Questionnaire [81]	Appearance evaluation subscale	Most similar to the other rating scales in other studies	Stage of pregnancy not stated, Non-pregnant control
Ruggieri et al. (1979) [59]	Moderate	Body Cathexis Scale [74])	Body Cathexis Scale	Only one scale was used in this study	Trimester 3, Non-pregnant control
Skouteris et al. (2005) [12]	High	Body Attitudes questionnaire (BAQ; [75]; Pregnancy Figure Rating Scale (PFRS; [12]; Physical Appearance Comparison Scale (PACS; [15]	Body Attitudes questionnaire (feeling fat subscale)	BAQ is more relevant to pregnancy because the focus is on body satisfaction. PACS is a comparison rather than absolute measurement. PFRS is only relevant to pregnancy, not control and is therefore not comparable with the other studies	Retrospective measure of 3 months pre-pregnancy Early trimester 2 (16–23 weeks) Late trimester 2/early trimester 3 (24–31 weeks) Late trimester 3 (32 + weeks)

Note: *Measurements excluded from analysis (explained in Data analysis section)

Details of bibliographic data about each study, demographic data, moderators, study design and points of assessment, gravidity (whether the current pregnancy is the first or a subsequent pregnancy), body image dissatisfaction measure, results and data analysis processes were taken from each eligible study by two authors blindly, and a sample checked for inter-coder agreement. Some studies reported data from multiple measurement tools; in these cases the most relevant to body image dissatisfaction in pregnancy were chosen so that one single figure representing body dissatisfaction was used for the meta-analysis. This ensured that all data points for analysis were independent, therefore a simple meta-analysis was considered most suitable. This is outlined and explained in Table 3.

### Data analysis

To allow a single data point for each study, when within participant studies analysed multiple results from different gestational stages for the pregnant data, we took a simple mean body dissatisfaction score to represent the body dissatisfaction in the pregnant sample when there was no attrition [12, 64] or no attrition was reported [56]. When attrition was reported, a weighted mean of the data points gathered during pregnancy [31] was calculated and used. This allowed a more direct comparison between all the studies so that they all include a single pregnancy and a single non-pregnancy measure, and ensured that all measures were independent [85].

Some measurements were excluded from the meta-analysis: Clark and Ogden [60] used both concurrent and prospective data, but only the concurrent data was used for the meta-analysis, as it is recommended that within participant designs should be avoided for meta-analyses if a between participants design effect size can be used [86, 87]. Postpartum measurements were excluded from Chan et al. [31] and Lombardo’s [61] studies. Gough [64] collected data at 20 weeks and 34 weeks gestation, although only data from 20 weeks was analysed as this was considered more comparable with the majority of the other studies; likewise McCarthy’s [70] measure at 32 weeks was more comparable with other studies than the measurement at 10.5 weeks and so the 32 weeks data was used (see Table 3, indicated with *).

The data were continuous for each study, so standardised mean differences were calculated. Cohen’s D [88] was chosen as the appropriate effect size because it is effective when using standardised mean differences [89]. The main effect was derived from the mean difference between the pregnant and non-pregnant scores, regardless of whether this was repeated measures or independent measures designs. Small, medium and large effect sizes were represented by Cohen’s guidelines [88] of ≤ 0.2, 0.5 and ≥ 0.8 respectively, although treated with some flexibility [90]. Effect sizes for within and between designs were calculated using the same method. There is disagreement within the literature whether within groups designs should use a different formula or method than independent samples to calculate effect sizes [91]. However to do this, correlational data or f/t values would be required, which were not available in most studies, so according to the generalizable effect size estimate viewpoint, it was deemed acceptable to use the same calculation for the between and the within subjects designs, as they are conceptually similar [91]. Random effects analysis was more appropriate than a fixed effects meta-analysis as the type of measures, procedures and contexts used in the studies were heterogenous [92], as well as initial inspection of the data showing statistical heterogeneity [93]. Weighting was undertaken using the standardised mean difference and pooled standard deviation, giving the inverse variance of each study. All analyses were conducted in R 4.2.2 using the metafor package [94], specifically a random effects meta-analysis and moderated meta-analysis were undertaken. Tau’s test of heterogeneity was applied, and funnel plot asymmetry was tested using Egger’s regression test, a funnel plot was applied to the data. The full data set, quality assessment and R script are available in the Open Science Framework (see https://osf.io/dxf8z/).

## Results

Seventeen studies (K = 17, with 17 effect sizes) were analysed, ranging from the years 1979–2022, comprising 5200 responses from women when they were pregnant and 4172 responses from women when they were not pregnant. A total of 7630 independent women were included in the studies; 1742 in the four within participant design studies; plus 6016 women in the thirteen between measures design studies, of which 3586 were pregnant and 2430 were not pregnant. Sample sizes varied from 38 to 1792, and ages varied from M = 19.43 years to M = 32.4 years, with most studies having an average age around 30 years. Table 4 shows the demographic details for the studies. Quality assessment indicated that all 17 studies were of high enough quality to be included in the analysis, with most being rated as having moderate or high quality, although most studies had some minor or substantial flaws (Table 2).

**Table 4:** Summary of demographic details of each study included in the meta-analysis

Study authors	Age; M(SD)		Gravidity	Country
	Pregnant	Non-pregnant	Primigravid (First pregnancy)	
Chan et al. (2020) [31]	31.97 (4.10)**		52%	China (Hong Kong)
Clark and Ogden (1999) [60]	27.96 (4.75)	26.56 (3.24)	100%	England
Crossland et al. (2022)* [55]	32.2 (4.9)	32.7 (5.44)	55%	UK
Davies and Wardle (1994) [57]	30.13 (5.23)	29.18 (6.16)	Average 0.8 children	England
Duncombe et al. (2008) [56]	31.7 (3.7)**		45.10%	Australia
Fuller-Tyszkiewicz et al. (2020) [65]	29.76 (3.26)**		41%	US and UK
Gough (1998) [64]	31 (3.8)**		66%	England
Harrison et al. (2019)* [63]	19.43 (2.85)	19.97 (2.72)	Information not provided	Canada
Inanir et al. (2015)* [36]	24.8 (5.1)	26.23 (4.9)	Information not provided	Turkey
Lombardo (2001)* [61]	28.4 31 (3.41)**		100%	USA
Loth et al. (2011)* [14]	25.83 (SD not stated)**		44.1%	USA
McCarthy (1998)* [70]	26.5	30	100%	USA
Meireles et al. (2021) [66] and Hudson et al. (2021)* [67]	29.00 (4.77)	20.77 (2.30)	55.40%	Brazil
Pascoal et al. (2019) [62]	31.93 (3.45)**		58.10%	Portugal
Pieta et al. (2021)* [58]	31.94 (4.6)	31.05 (8.57)	29.8%	Poland
Ruggieri et al. (1979) [59]	30 (SD not stated)**		Information not provided	Italy
Skouteris et al. (2005) [12]	31.63 (3.44)**		49%	Australia

Note. *Scores inverted so that high scores in all studies represent high body dissatisfaction. ** studies reported combined age data for the two groups

In nine studies, a high score on the scale used equated to high body image dissatisfaction, and in the other eight studies high scores equated to low body image dissatisfaction (reported in Table 4). The latter scales were inverted so that all the results showed a high score equated to high body image dissatisfaction. All further analyses used this standardised presentation of high scores equating to high body image dissatisfaction (studies identified in Table 4). A positive effect size indicates that the pregnant subgroup had higher scores and therefore higher body image dissatisfaction compared to the non-pregnant subgroup.

**Fig. 2:**
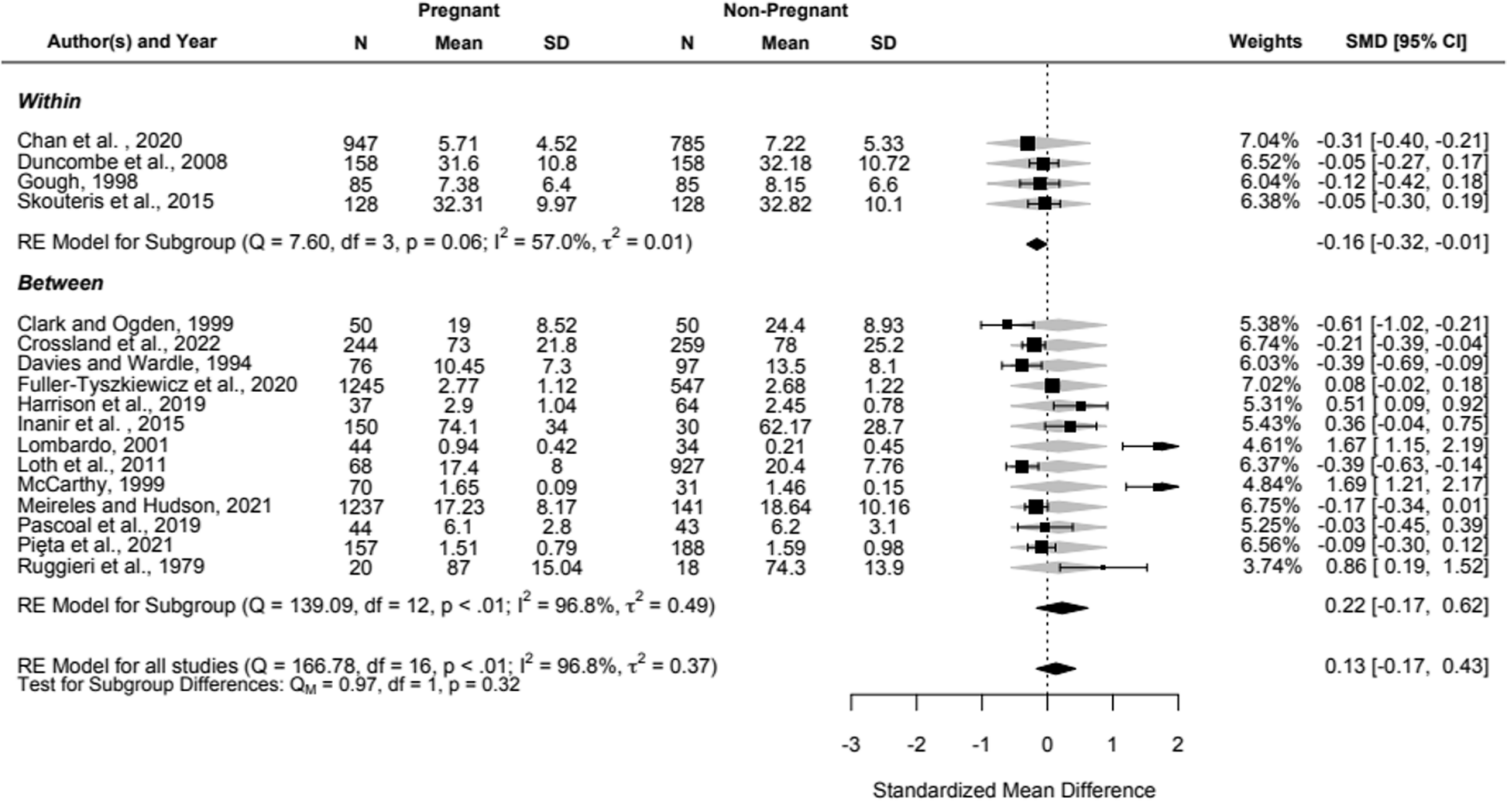
Forest plot for the pooled and weighted effect sizes (Cohen’s D) of changes in body image dissatisfaction Positive score relates to an increased report of body image dissatisfaction. Note: Scores for 14, 36, 55, 58, 61, 63, 66 and  xx/69 are reversed to align with other studies so high numbers denote high body image dissatisfaction

The meta-analysis summary results and 95% confidence intervals relate to the standardised mean difference between pregnant body image dissatisfaction and non-pregnant body image dissatisfaction. A random effects model was fitted to the data because of heterogeneity of measures and methodology in the studies [89]. This is also demonstrated by the Tau^2^ test of heterogeneity, which suggests that there is large variation in outcomes between the studies (Tau^2^ = 0.37, *p* < 0.001). Figure 2 shows the forest plot for the pooled changes in body image dissatisfaction in the pregnant samples compared with the non-pregnant comparisons (k = 17), using a simple meta analysis, displaying the averaged difference in means between the pregnant and non-pregnant participants in each study. The weighted outcome did not differ significantly from zero (z = 0.86, *p* = 0.39) and the analysis showed an overall effect size of 0.13. The confidence intervals range from -0.17 to 0.43, which crosses the zero point, therefore supporting the null hypothesis.

Potential moderator analyses were considered, including age, BMI, gravidity and mental health. However, due to lack of number of studies and lack of/inconsistent reporting of details, analyses were not possible on most potential moderators apart from study design (see Table 5). A moderated meta-analysis was conducted to investigate if the design of the study affected outcomes as design was reported in all papers. The results were non-significant (*p* = 0.17), suggesting that the use of independent samples (overall effect size = 0.22) or repeated measures samples (ES = -0.16) did not affect outcomes, however, due to there being only four with repeated measures samples these results should be interpreted with caution. Gestation was also reported in all but two papers [14, 58]. When gestation was reported, this was done in many different ways (e.g. dividing into trimesters [31, 36, 65], dividing into three groups that did not correspond to trimesters [12, 56], or only recruiting in a specific trimester/ gestational age [57, 59, 60, 63, 64, 70], such that analysis of gestation as a moderator was not possible. One notable participant variable that was hypothesised to affect body image dissatisfaction in pregnancy is gravidity. However, only three studies analysed the effect of gravidity [31, 55, 64]; two of these studies found that women in their first pregnancy (primigravidae) have lower body dissatisfaction than multigravidae (M = 5.76 / 6.01 [31] and M = 139.2 / 143.8 [55] for primigravidae and multigravidae, respectively), whereas the third study [64] found primigravidae had slightly higher body dissatisfaction (M = 9.61) than multigravidae (M = 7.01). Due to the other fourteen studies not reporting separate means for gravidity (N=8; [12, 14, 56–58, 62, 65–67]), not reporting gravidity (N=3; [36, 59, 63]), or only testing primigravidae (N=3; [60, 61, 70]) no statistical analysis was possible within this meta analysis.

**Table 5:** Availability of analyses according to moderator variables

	Age	Gravidity	Mental health	Pre-pregnancy BMI	Gestational weight gain/current BMI	Trimester of pregnancy
Chan et al. (2020) [31]	X	**Y**	**Y**	X	X	**Y**
Clark and Ogden (1999) [60]	X	X	X	X	X	X^**2**^
Crossland et al. (2022) [55]	X	Y	X	X	X	Y*
Davies and Wardle (1994) [57]	X	X	X	X	X	X^**2**^
Duncombe et al. (2008) [56]	X	X	X	X	X	X^**1**^
Fuller-Tyszkiewicz et al. (2020) [65]	X	X	X	X	X	**Y**
Gough (1998)* [64]	X	**Y**	X	X	X	X^**2**^
Harrison et al. (2019) [63]	X	X	X	X	X	X^**2**^
Inanir et al. (2015) [36]	X	X	X	X	X	**Y**
Lombardo (2001)* [61]	X	X	X	X	X	X
Loth et al. (2011) [14]	X	X	X	X	X	X^3^
McCarthy (1998)* [70]	X	X	X	X	X	X^**2**^
Meireles et al. (2021) [66] and Hudson et al. (2021)** [67]	X	X	X	X	X	X
Pascoal et al. (2019) [62]	X	X	X	X	X	X
Pieta et al. (2021) [58]	X	X	X	X	X	**Y**^3^**
Ruggieri et al. (1979) [59]	X	X	X	X	X	X^**2**^
Skouteris et al. (2005) [12]	X	X	X	X	X	X^**1**^

Note: X denotes analysis of the moderator was not possible. Y denotes that analysis of the moderator would be possible. *data available on OSF **data provided on request from author. ^**1**^Divided participants into 3 groups that did not correspond with trimesters. ^**2**^Used participants from only one trimester or one point in pregnancy. ^3^Stage of pregnancy not stated

Another notable moderator variable that was hypothesised to relate to body image dissatisfaction is mental health status. Only 5 studies analysed this in relation to body image dissatisfaction [12, 31, 56, 66, 67, 70] so could not be analysed statistically. Only one study considered mental state as a direct moderator for the impact of pregnancy on body image dissatisfaction, finding that the correlation between depression and body dissatisfaction is stronger during pregnancy (0.15) than when not pregnant (0.09), with a similar pattern for anxiety (0.09/0.08 respectively; 31). Other studies [56, 66, 67] found a correlation between depression and body image dissatisfaction in pregnancy (0.38, 0.43 respectively), although did not take a non-pregnancy measure of depression [56] or compare depression directly as a moderator in the non-pregnant sample [66, 67] to allow a comparison. Further studies only reported depression as a moderator for the whole group (0.47 and 0.21-0.52; [12, 70]). Even fewer studies reported the relationship between BMI and body image dissatisfaction [12, 36, 66, 67], with none directly comparing whether the relationship between these two factors differed between the two groups.

**Fig. 3:**
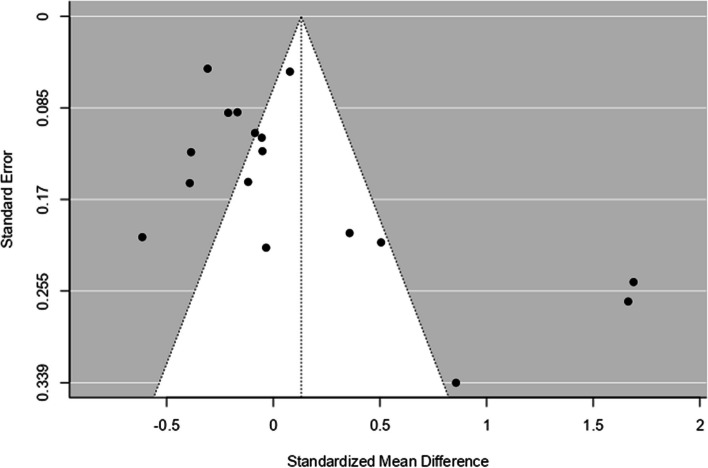
Funnel plot displaying effect size and standard error

Interestingly on inspection of the forest plot, 2 clear outliers were identified with very high effect sizes [61, 70]. The main analysis was conducted again without the outliers, which still showed a non-significant result (*p* = 0.202). A funnel plot of the outcome measures was calculated (Fig. 3). Egger’s regression test indicated some asymmetry (*p* = 0.0011), whereby smaller studies that did not show a group difference may not have been published, although many other reasons could also explain this funnel plot asymmetry, such as methodological differences between studies gleaning different effect sizes [95].

## Discussion

This systematic review and meta-analysis of 17 studies compared body image dissatisfaction in pregnant women (total 5200 responses) to women who were not pregnant (total 4172 responses), across 13 between participant design studies [14, 36, 55, 57–60, 62, 63, 65–67, 70] and 4 within participant design studies [12, 31, 56, 64]. The main statistical analysis on a group level showed that women’s body image dissatisfaction was not statistically different during pregnancy compared with when not pregnant. However results from Tau’s test of heterogeneity and inspection of individual studies indicated large variation in the results, with some studies demonstrating that on a group level body image dissatisfaction is lower when pregnant [14, 31, 55, 57, 60, 64, 66, 67], others showing that body image dissatisfaction is higher when pregnant [36, 59, 61, 63, 70], and other studies finding no significant group difference [12, 56, 58, 62, 65]. This may suggest that by synthesising results from several studies, the outcome has regressed to the middle ground.

Conflicting theories explaining the potential impact of pregnancy on body image dissatisfaction suggested that pregnancy represents either a deviation or liberation from social ideals, thus predicting increased and decreased body image dissatisfaction, respectively. The current results, reveal no overall pattern, suggesting that neither argument is strongly supported by the synthesis of the existing literature. Inspection of the individual studies in the context of the systematic literature review indicated that the one consistent pattern was that of heterogeneity. Individuality of experience appeared to be paramount rather than time frame of pregnancy [12], highlighting genuine differences in perinatal experience, as suggested by previous qualitative data (e.g. 11,49), and warning against generalisations across cohorts or assumptions that all pregnant women experience similar feelings towards their changing body [96]. As well as representing genuine variation between individuals and between studies, the overall null result of the meta-analysis, and the heterogeneity of the effect sizes and variances within the studies is likely to reflect differences in methodology, measurement devices and stages of pregnancy assessed.

These results could be due to many individual differences within pregnancy, which may be explained by mediational processes such as those described in the thin-ideal internalisation model [10], such as how much an individual internalises social pressures. Internalisation involves the active endorsement of societal values whereby they influence a person’s attitudes and / or behaviour—in this case their attitudes towards the external appearance of their body and behaviours such as eating behaviours [97], which also occurs during pregnancy [98]. Research has found internalisation of the thin ideal to be more important in influencing body image dissatisfaction than mere awareness of cultural pressures to be thin [97]. It may be the case that some pregnant women are passively aware of thin ideals, but do not internalise them, which could at least partly explain the inter-study differences found. However the importance of supporting a healthy pregnancy and developing fetus may override other cognitions about themselves and their body [99].

The synthesised results could also depend on the relative importance that each individual places on the function of the body and physical appearance [100], whereby if women are more focussed on body functionality they may be less concerned with how the body looks during pregnancy [19]. Further research also differentiates a clear maternal ideal, which focuses on the body’s functionality to provide specifically for the fetus [101] and embark on the role of motherhood as an entirely different concept to the non-maternal body ideal. Evidence also suggests the role of self-acceptance (unconditionally accepting oneself despite acknowledging any flaws; [102]) is important in body image satisfaction during pregnancy [103], and for mental health [49]. As pregnancy can affect a woman’s identity [104], her level of acceptance of her physical and psychological attributes can protect her well-being. This can fluctuate during the perinatal period [104] possibly because of such vast physiological and psychological changes in this time, which may have an impact on body image dissatisfaction [103]. Further research measuring internalisation of body ideals, focus on functionality and self-acceptance could be undertaken to distinguish their relative roles in body dissatisfaction during pregnancy.

In terms of methodological factors, it is important to consider the design of the study, as previous research has suggested that many psychosocial [12] and demographic factors [105], and parental status [55] differ between pregnant and non-pregnant samples, making comparisons between two different groups less useful as they are not comparing like with like. However, using one sample in a study and therefore asking pregnant women to retrospectively recall feelings towards their body from several months prior to pregnancy could be problematic due to a “maturation threat” to their recall [106] whereby their feelings to their body change over time, which affects their recall of their pre-pregnancy state. This may be particularly the case in pregnancy as the individual is experiencing such vast changes in their body, and also potentially their body image. There is also the possibility that their body image dissatisfaction prior to pregnancy may not reflect their feelings towards their usual non-pregnant body, particularly if they were trying to be healthy during the preconception stage [107], or if they had experienced previous miscarriages [108] or problems conceiving [109].

Being able to analyse other potential moderators could have provided more insight into the discrepancies in results between different studies, however there were not enough studies that fitted all analysis criteria to be able to do this effectively, and many of the studies did not report information that could have been moderators. For example pre-pregnancy or pregnant BMI was only analysed as a potential moderator to body image dissatisfaction in three papers [12, 36, 66, 67], and in most papers was either reported but not analysed (*N* = 8; [14, 31, 36, 56, 57, 60, 64, 65]) or not reported at all (*N* = 6; [55, 58, 59, 61–63, 70]). Likewise mental health status was analysed as a factor that could affect body image dissatisfaction in five papers [12, 31, 56, 66, 67, 70], but in most was either reported but not analysed or not able to be analysed due to how data was presented (*N* = 5; [12, 56, 58, 66, 67]) or not reported at all (*N* = 11; [14, 36, 55, 57, 60, 61, 63–65]). Few research papers distinguished between gravidities when investigating body image dissatisfaction in pregnancy, and even fewer (*N *= 1 from this review) distinguish between women in the control group who have already had children and those who haven’t, which has been found to have an impact on body image dissatisfaction [55]. The difference in body image dissatisfaction between women experiencing their first and subsequent pregnancies could be due to ongoing bodily changes from previous pregnancies or due to social and role changes from being parents, indicating that gravidity may be an important factor to investigate and understand. Future research into body image dissatisfaction in pregnancy would benefit from this distinction. More detailed and complete reporting of procedures and data would help progression in this field of research. Although it is important to note that most of the study designs were suitable for the individual study aims, synthesis of the literature, such as the current meta-analysis, help to identify important factors that should be considered in future research.

### Strengths and limitations

There are various strengths and limitations of meta-analyses, and of the current meta-analysis. The screening process for this study was meticulously and objectively undertaken blindly by two authors (AC and CP), with very few disagreements; any that occurred were resolved through closer inspection and consideration of the available data. A quality assessment was conducted blindly, following JBI guidelines [68] and checked by all authors. No quality assessment tool was available that fit the exact type of study as there was no direct intervention, but as there were control (non-pregnant) participants, cross-sectional guidelines were deemed the most appropriate. This meant that some quality ratings were not relevant, or lower than one might expect, however this was not considered to mean the studies were of poor quality (e.g. [65]). More appropriate tools for analysing risk of bias in non-intervention studies would be useful for similar reviews and meta-analyses in the future.

A weakness with the current study is that data measured at different timepoints in pregnancy were combined into one pregnancy measure and compared with the non-pregnancy measure. This was done because studies reported gestation in very different ways, that were incomparable, for example some studies reported by trimester and used one measure per trimester [31, 36, 65–67], others used participants all in one trimester [57, 60, 63, 64, 70], further studies used samples from early and late trimester two, and trimester 3 [12, 56]. Others did not report gestational age [14, 58] or used an unconventional measurement [59]. This is an issue because body image dissatisfaction is thought to vary during gestation [36], and so using an ‘average’ or choosing one time point in pregnancy could be subject to transient errors [110]. For example in a systematic review of 10 qualitative studies exploring body image in the perinatal period, women in the early stages of pregnancy reported feeling fat/frumpy because at that stage the expectant mother is not showing a typical pregnant body [20]. But once the ‘bump’ shows, it was reported that women often feel more confidence in their body shape [12, 31]. Whether using snapshot studies, or averaging the data across the pregnancy, a fair representation of body image dissatisfaction in pregnancy may not have been presented.

There are also several strengths and limitations of the literature gathered within the systematic review and meta-analysis. The nature of a meta-analysis acknowledges the risks of publication bias and tries to reduce it by synthesising studies, testing for funnel plot asymmetry, and searching the grey literature. The two outlying studies [61, 70] identified in the meta-analysis were from the grey literature, and therefore the high effect sizes reported in these studies may have been due to lack of methodological rigour [93]. Although Egger’s regression indicated funnel plot asymmetry, which could be interpreted as publication bias [93], that is unlikely to be the case in the current study, because unpublished studies were screened and included. The funnel plot and original data suggest that studies with significant results in both directions and non-significant results were published in the area. This means that we could assume that the articles used give a valid account of the variety of changes in body image dissatisfaction between pregnant and non-pregnant women, although it is important to acknowledge that some relevant studies may not have been selected in the initial systematic search if the title or abstract did not contain the chosen search terms.

One difficulty in directly comparing and synthesising research in the field of body image dissatisfaction is a lack of clarity and consistency of the construct itself. In much of the literature the terms body image dissatisfaction and body dissatisfaction appear to be used almost synonymously, however, each of these terms differs in subtle ways. For example, in pregnancy one may be dissatisfied with their body image (how it looks) but satisfied with their body (as it safely develops a fetus) or vice versa. Likewise when researchers use the term “body image dissatisfaction”, there is disagreement over a working definition which varies from “negative thoughts and feelings that people may have about their bodies and appearance [111, 112] to more specifically, a “negative attitude towards one’s own body resulting from a perceived discrepancy between the actual body image … and the ideal body image” ([113]; p158). Some definitions of body image dissatisfaction (for example the latter here) are less appropriate to use when discussing body image dissatisfaction during pregnancy.

Furthermore a woman experiences many specific changes to their appearance and function that are unique to pregnancy, so scales that do not pick up these intricacies may not identify subtle differences in body image dissatisfaction specifically during pregnancy [42], possibly reducing validity of results from the pregnant samples [105]. For example, evidence suggests that feelings towards different parts of the body differ, such as women reported accepting weight gain on the stomach, hips and buttocks but felt less positive about weight in other areas such as arms and face [114]. This indicates that scales relating to the body as a whole may not capture these internal inconsistencies. Indeed the sensations of carrying a fetus are different to those of carrying ‘fat’ and therefore scales that do not differentiate these feelings may not identify valid feelings in pregnancy [12]. Likewise in a widely used and validated measure of body image dissatisfaction (Eating Disorder Inventory, EDI; [71]) one subscale is entitled ‘Drive for Thinness’, which would not necessarily be relevant to pregnant women. One commonly used term in body image satisfaction scales is ‘fat’ or ‘fatness’, for example the Body Attitudes Questionnaire includes a ‘feeling fat’ subscale, with high scores accompanying high negative scores in other subscales. This makes the assumption that being or feeling fat is a negative thing, whereas during pregnancy putting on a healthy amount of weight is recommended [115]. Therefore, some of the scales that refer to ‘fat’ as a negative connotation may not be suitable or valid for use amongst pregnant women [105]. More research is needed into validating scales equally for pregnant and not pregnant women to ensure that they are capturing the same feelings in both populations to avoid measurement error and allow directly comparable results between studies.

A further concern with the analysed studies is that overall the samples were relatively homogenous, with most including well educated, mainly white samples. Papers that reported relationship status tended to report a high majority of participants were in relationships, with slightly lower proportions of non-pregnant women in relationships (when reported). Clinical samples were excluded because their pattern of weight gain [116], and eating habits during pregnancy are different to non-clinical populations [117] and therefore any changes in body image dissatisfaction would be not directly comparable with a non-clinical population. Additionally eating disorder symptoms are qualitatively different to merely extreme body image dissatisfaction [118] and so exclusion of this group excluded the potential for many variables that may not have been relevant to the general population. What’s more, the inclusion criteria dictated that all the included papers were in English, which excluded foreign language papers. This may have the consequence of Western bias because expectations of female bodies can differ vastly in different countries and cultures [119], as well as expectations of pregnancy and motherhood. Most of the studies gathered data in Europe [55, 57–60, 62, 64, 65], the Americas [14, 61, 63, 65–67, 70] or Australia [12, 56], with only two of the studies conducted in a non-Western country, specifically China [31] and Turkey [36]. These two studies produced opposing results, with one finding that body dissatisfaction is lower in pregnant women than non-pregnant in China [31], and the other showing opposing results in Turkey [36], despite body ideals being similar in China [8] and Turkey [9] to Western cultures. This further emphasises the need to undertake research into body image dissatisfaction in pregnancy using more diverse samples. We should also consider how expectations of women’s bodies and behaviours during pregnancy are heavily entrenched in cultural ideals, which could suggest that deviance away from cultural norms should be considered as much as measuring body image satisfaction itself.

### Implications

The mixed outcomes from the systematic review and meta-analysis suggest that rather than attempting to generalise across pregnancy, the individual experience should be considered in depth to understand exactly what makes a pregnant woman feel as they do about their changing body, and the important implications of their bodily experience. The consideration of moderators that could affect body image dissatisfaction during pregnancy is important, such as BMI (current and pre-pregnancy), gravidity (or already having children), mental health status and the country the study was conducted in, amongst others. This then raises the, possibly more important, issue that health professionals and expectant mothers should focus on talking about their body, whether they have concerns or confidence, and to deal with each pregnant woman on a case by case basis. Perinatal wellbeing can have many consequences for the mother and baby, both physiologically and mentally, as well as their relationship with each other, so is an important area of focus for research and in clinical practice. The current analysis highlights the need for body image satisfaction scales to be developed and validated for use in both pregnant and non-pregnant populations, as well as highlighting the importance of reporting full data for the purposes of open science and synthesis of data.

## Conclusions

Our analysis reveals, on a group level, no statistical difference in body image dissatisfaction between pregnant and non-pregnant women. Yet a closer look at the data exposes a range of experiences and attitudes to the body among pregnant women, as well as inconsistencies in research methods and measurement. Body image dissatisfaction in pregnancy seems to be a combination of many complex factors related to the individual experience of each pregnant woman, and therefore more research is needed to understand the explanatory factors underpinning the variation in changes in body image dissatisfaction during pregnancy compared with when not pregnant.

### Supplementary Information

**Additional file 1. **

## Data Availability

The datasets generated and/or analysed during the current study are available on OSF: https://osf.io/dxf8z/.
