# Supplementary material for: Comparing body image dissatisfaction between pregnant women and non-pregnant women: a systematic review and meta-analysis
Source: BMC Pregnancy Childbirth. 2023 Oct 4;23:709. doi: 10.1186/s12884-023-05930-w (PMC10548696; doi:10.1186/s12884-023-05930-w)

Additional files

Additional file 1: Protocol

**A meta-analysis of the effect of pregnancy on body image dissatisfaction**

**Protocol (Based on Cochrane recommendation)**

**Brief background**

Pregnancy is a unique time in a woman’s life, during which her body undergoes significant physical changes over a relatively short period of time. Many women experience physical issues such as fatigue (Chien & Lo, 2004), pain (Vermani, Mittal, & Weeks, 2010; Kovacs et al., 2012) and urinary incontinence (Glazener, et al. 2006) as well as physical changes such as their abdomen and breasts increasing in size (Rasmussen et al., 2009), their posture altering (Bullock et al., 1987) and their hair and skin changing (Muzaffar et al., 1998). Many of these bodily changes are in direct conflict to Western social ideals of female body appearance (Greer & Davis, 1984), but are also signs of adjusted functionality of the body and the associated positive feedback (Clark et al., 2009).

There are conflicting results in the literature, with studies revealing both an improvement in body satisfaction in pregnancy (Clark et al., 2009; Loth et al., 2011) and a worsening (Brown, Rance, & Warren, 2015; Inanir et al., 2015; Skouteris et al., 2005). This disagreement in the literature could be due to design differences in studies, for example some studies employ a within-subjects design, while others use a between-subjects design. Further differences could be due to some not testing for moderator variables like parity or pre-pregnancy status like BMI.

**Objectives**

The objectives of this systematic review are to assess the existing empirical data to conclude what effect pregnancy has on body satisfaction, and to consider the factors that might contribute to body satisfaction in pregnancy, in order to explain why some studies find an increase in body satisfaction and others a decrease. If the extracted data from the included studies allow, a meta-analysis of the data will be undertaken.

This is an important field to understand because antenatal body image concerns are linked to negative maternal and infant outcomes, including postpartum depression, low infant birth weight, and lower rates of breastfeeding (Brown, Rance & Warren, 2015; Conti, Abraham & Taylor, 1998; Scmied & Lupton, 2001), reduction in attachment (Kirk and Preston, 2019) and short and long term mental ill-health (Roomruangwong et al., 2017).  If body image dissatisfaction during pregnancy is understood interventions could be used early in pregnancy that could counteract the potential negative outcomes of body image dissatisfaction.

**Research Questions**

What impact does pregnancy have on body satisfaction?

Is any effect mediated by factors related to pre-pregnancy or antenatal physiological and psychological status?

**Methods**

**Eligibility Criteria**

- Study characteristics
  - Prospective studies that assess women pre-pregnancy and during pregnancy (within subjects)
  - Concurrent studies that compare pregnant and non-pregnant women (between subjects)
  - Exclude case studies and qualitative studies due to the proposal of meta-analytical data analysis
- Sample characteristics
  - Pregnant women
  - Exclude samples with known eating disorders
  - There are no exclusions on the country of origin
- Outcome characteristics
  - Studies assess body (dis)satisfaction
  - Quantitative measures of body satisfaction
- Article characteristics
  - Written in English
  - Reported sufficient data (or could be provided by authors)

**Databases**:

The following databases of published articles will be searched:

- Scopus,
- Psychinfo,
- Web of science,
- Pubmed,
- Cochrane library,
- Embase

These were chosen based on the databases outlined in various systematic reviews on the topic.

The following databases of unpublished and preprint articles will be searched:

- Open Grey
- PsycEXTRA/EBSCO
- OATD
- EThOS,
- Open Thesis,
- ProQuest Dissertations and Theses,
- WorldCat,
- DART-Europe E-theses Portal,
- Register of Commonwealth Research,
- Trove: the database of the National Library of Australia
- PsyArXiv,
- MedArXiv

The following databases of key research funders will be searched to identify end of award reports:

- RCUK Gateway to Research x
- NICHD – NIH Research Portfolio Online Reporting Tools (RePORT) x

Additionally we will search ResearchGate and Academia.Edu by named authors of eligible

studies. We will scan the included studies listed in systematic and non-systematic reviews to identify any further studies.

Once we have a list of candidate studies we will also contact researchers in the field to ask for suggestions of additional relevant published and unpublished studies that we might have missed in our searches.

The search terms used will be:

TITLE-ABS-KEY  ( pregnan*  OR  prenatal  OR  antenatal  OR  *gravid*  OR  *parous*  OR  gestation*  OR  perinatal )  AND  ( "body image"  OR  "body satisfaction"  OR  "body dissatisfaction"  OR  "body concern"  OR  "body preoccupation"  OR  "body attitude"  OR  "body image disturbance"  OR  "body image distortion" )  AND NOT  ("case study"  AND NOT  animal )

These search terms were chosen based on those found in various systematic reviews on the topic. If we detect additional relevant key words during any of the electronic or other searches, we will modify the electronic search strategies to incorporate these terms and document the changes.

Two reviewers (AH and CP) will independently review studies blindly at each stage. Any disagreements will be discussed, and if needed a third reviewer (LK) will be involved.

**Data extraction and analysis**

Two review authors (AH, CP) will independently scan the abstract, title, or both, of every record retrieved, to determine which studies should be assessed further. We will investigate all potentially relevant articles as full text. We will resolve any discrepancies through consensus or recourse to a third review author (LK). We will present an adapted PRISMA (Preferred Reporting Items for Systematic Reviews and Meta-Analyses) flowchart of study selection.

Information on the following elements will be extracted and recorded in a database (this list is a proposal, and is likely to change according to the data that is available in studies during extraction):

- Bibliographic data (title, journal, authors, year)
- Population
- N and attrition
- Mean age of sample
- Country
- Parity (% primiparous)
- Pre-pregnancy BMI
- Pregnancy BMI
- Gestational weight gain
- Relationship quality
- Mental health measurements
- Study type (within or between subjects)
- Number of times that body satisfaction was assessed
- Points of assessment (pre-pregnancy, 1st, 2nd, 3rd trimester or combination)
- Body satisfaction measure(s)
- Pre-pregnancy body satisfaction measure (retrospective or prospective)
- Data analysis - how was data analysed
- Results
- Mental health status (before and during pregnancy)
- Assessment of risk of bias, including:
  - Selection bias.
  - Blinding (performance bias and detection bias)
  - Incomplete outcome data (attrition bias).
  - Selective reporting (reporting bias).

The data extraction sheet will be piloted on a small number of studies before progressing to full data extraction. It will be extracted in a standardised template in a data coding sheet using Excel.

**Potential moderators:**

Potential moderators of the outcomes will be investigated. Below are some potential moderators; this list will be adjusted according to the initial data extraction:

- Type of study design: prospective studies (comparing the same women before pregnancy and during pregnancy) and cross-sectional (comparing pregnant and non-pregnant women).
- Pre-pregnancy BMI
- Parity
- Mental health status (before and during pregnancy)
- Gestational weight gain
- Age

**Proposed Analyses**

1. Main effect of pregnancy on body satisfaction
2. Moderator analyses (others may become apparent after studies are considered in depth):
   1. Categorical:
      1. Type of design (between/within)
      2. Trimester when BS assessed (1st, 2nd, 3rd)
      3. Parity (primiparous, multiparous)
      4. Publication status (published, unpublished)
   2. Continuous
      1. Mean gestational weight gain
      2. Mean pre-pregnancy BMI

3.Sensitivity analyses

Assess for statistical outliers  based on z-values in each analysis and remove. Additionally I will consider whether quality of study and the type of pre-pregnancy measure (retrospective or concurrent) impacted upon the results.

4.Publication bias

- Funnel plot

**Assessment of risk of bias in included studies**

Two review authors (AH, CP) will assess the risk of bias of each included study independently. We will resolve disagreements by consensus, or by consultation with a third author (LK). We will assess risk of bias using the Cochrane Collaboration’s tool for assessment of risk of bias (Higgins 2011a; Higgins 2011b).

We will assess various criteria in this assessment (list is not exhaustive at this stage):

• Selection bias.

• Blinding (performance bias and detection bias)

• Incomplete outcome data (attrition bias).

• Selective reporting (reporting bias).

• Other bias.

**Dissemination plans**

The meta-analysis will be submitted for review at a peer-reviewed psychology journal.

Additional file 2: Grey Literature search

- Open Grey
- PsycEXTRA/EBSCO
- OATD
- EThOS,
- Open Thesis,
- ProQuest Dissertations and Theses,
- WorldCat,
- DART-Europe E-theses Portal,
- Register of Commonwealth Research,
- Trove: the database of the National Library of Australia
- PsyArXiv,
- MedArXiv

Additional file 3: Eligibility criteria

- Study characteristics
  - Prospective studies that assess women pre-pregnancy and during pregnancy (within subjects)
  - Concurrent studies that compare pregnant and non-pregnant women (between subjects)
  - Exclude case studies and qualitative studies due to the proposal of meta-analytical data analysis
- Sample characteristics
  - Pregnant women
  - Exclude samples with known eating disorders
  - There are no exclusions on the country of origin
- Outcome characteristics
  - Studies assess body (dis)satisfaction
  - Quantitative measures of body satisfaction
- Article characteristics
  - Written in English
  - Reported sufficient data (or could be provided by authors)

Additional file 4: Data extraction

| **Title** |
| --- |
| **Journal** |
| **APA ref** |
| **Notes of interest** |
| **Population** |
| **N and attrition** |
| **Mean age of sample** |
| **Country** |
| **Parity (% primiparous)** |
| **Non-pregnancy BMI** |
| **Pregnancy BMI** |
| **Gestational weight gain** |
| **Relationship quality** |
| **Depression** |
| **Study type (within or between subjects)** |
| **Number of times that body satisfaction was assessed** |
| **Points of assessment (pre-pregnancy, 1st, 2nd, 3rd trimester or combination)** |
| **Body satisfaction measure(s)** |
| **Details of measurement** |
| **Retrospective pre-pregnancy body satisfaction measure?** |
| **Data analysis - how analysed the data** |
| **Descriptive data** |
| **Mean Pregnancy score** |
| **Mean non-pregnany score** |
| **T/F score** |
| **P value** |
| **Effect size measure** |

##

Additional file 5: JBI critical appraisal checklist

Moola, S., Munn, Z., Tufanaru, C., Aromataris, E., Sears, K., Sfetcu, R., Currie, M., Qureshi, R., Mattis, P., Lisy, K. and PF, M. (2019). Chapter 7: Systematic reviews of etiology and risk: The Joanna Briggs Institute; 2017. [Available online] <https://synthesismanual.jbi.global> [accessed 09/09/2022]

1. Were the criteria for inclusion in the sample clearly defined?

The authors should provide clear inclusion and exclusion criteria that they developed prior to recruitment of the study participants. The inclusion/exclusion criteria should be specified (e.g., risk, stage of disease progression) with sufficient detail and all the necessary information critical to the study.

2. Were the study subjects and the setting described in detail?

The study sample should be described in sufficient detail so that other researchers can determine if it is comparable to the population of interest to them. The authors should provide a clear description of the population from which the study participants were selected or recruited, including demographics, location, and time period.

3. Was the exposure measured in a valid and reliable way?

The study should clearly describe the method of measurement of exposure. Assessing validity requires that a 'gold standard' is available to which the measure can be compared. The validity of exposure measurement usually relates to whether a current measure is appropriate or whether a measure of past exposure is needed.

Reliability refers to the processes included in an epidemiological study to check repeatability of measurements of the exposures. These usually include intra-observer reliability and inter-observer reliability.

4. Were objective, standard criteria used for measurement of the condition?

It is useful to determine if patients were included in the study based on either a specified diagnosis or definition. This is more likely to decrease the risk of bias. Characteristics are another useful approach to matching groups, and studies that did not use specified diagnostic methods or definitions should provide evidence on matching by key characteristics

5. Were confounding factors identified?

Confounding has occurred where the estimated intervention exposure effect is biased by the presence of some difference between the comparison groups (apart from the exposure investigated/of interest). Typical confounders include baseline characteristics, prognostic factors, or concomitant exposures (e.g. smoking). A confounder is a difference between the comparison groups and it influences the direction of the study results. A high quality study at the level of cohort design will identify the potential confounders and measure them (where possible). This is difficult for studies where behavioral, attitudinal or lifestyle factors may impact on the results.

6. Were strategies to deal with confounding factors stated?

Strategies to deal with effects of confounding factors may be dealt within the study design or in data analysis. By matching or stratifying sampling of participants, effects of confounding factors can be adjusted for. When dealing with adjustment in data analysis, assess the statistics used in the study. Most will be some form of multivariate regression analysis to account for the confounding factors measured.


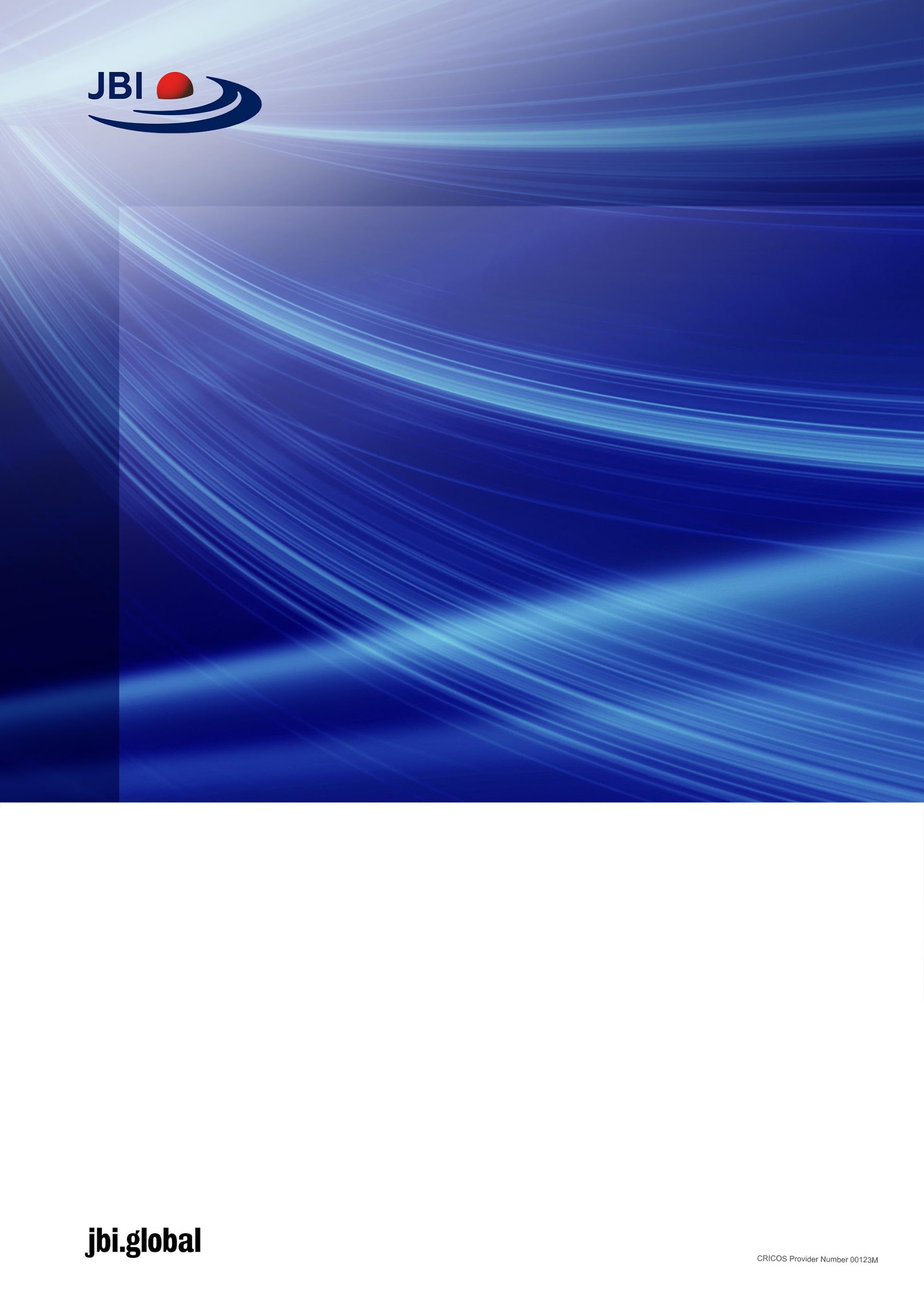
7. Were the outcomes measured in a valid and reliable way?

Read the methods section of the paper. If for e.g. lung cancer is assessed based on existing definitions or diagnostic criteria, then the answer to this question is likely to be yes. If lung cancer is assessed using observer reported, or self-reported scales, the risk of over- or under-reporting is increased, and objectivity is compromised. Importantly, determine if the measurement tools used were validated instruments as this has a significant impact on outcome assessment validity.

Having established the objectivity of the outcome measurement (e.g. lung cancer) instrument, it’s important to establish how the measurement was conducted. Were those involved in collecting data trained or educated in the use of the instrument/s? (e.g. radiographers). If there was more than one data collector, were they similar in terms of level of education, clinical or research experience, or level of responsibility in the piece of research being appraised?

8. Was appropriate statistical analysis used?

As with any consideration of statistical analysis, consideration should be given to whether there was a more appropriate alternate statistical method that could have been used. The methods section should be detailed enough for reviewers to identify which analytical techniques were used (in particular, regression or stratification) and how specific confounders were measured.

For studies utilizing regression analysis, it is useful to identify if the study identified which variables were included and how they related to the outcome. If stratification was the analytical approach used, were the strata of analysis defined by the specified variables? Additionally, it is also important to assess the appropriateness of the analytical strategy in terms of the assumptions associated with the approach as differing methods of analysis are based on differing assumptions about the data and how it will respond.

Quality analysis

|  | Were the criteria for inclusion in the sample clearly defined? | Were the study subjects and the setting described in detail? | Was the exposure measured in a valid and reliable way? | Were objective, standard criteria used for measurement of the condition? | Were confounding factors identified? | Were strategies to deal with confounding factors stated? | Were the outcomes measured in a valid and reliable way? | Was appropriate statistical analysis used? |
| --- | --- | --- | --- | --- | --- | --- | --- | --- |
| **Chan et al. (2020)** |  |  |  |  |  |  |  |  |
| **Clark and Ogden (1999)** |  |  |  |  |  |  |  |  |
| **Crossland et al (2022)** |  |  |  |  |  |  |  |  |
| **Davies and Wardle (1994)** |  |  |  |  |  |  |  |  |
| **Duncombe et al. (2008)** |  |  |  |  |  |  |  |  |
| **Fuller-Tyszkiewicz et al. (2020)** |  |  |  |  |  |  |  |  |
| **Gough (1998)*** |  |  |  |  |  |  |  |  |
| **Harrison et al. (2019)** |  |  |  |  |  |  |  |  |
| **Inanir et al. (2015)** |  |  |  |  |  |  |  |  |
| **Lombardo (2001)*** |  |  |  |  |  |  |  |  |
| **Loth et al. (2011)** |  |  |  |  |  |  |  |  |
| **McCarthy (1999)*** |  |  |  |  |  |  |  |  |
| **Meireles and Hudson (2021)**** |  |  |  |  |  |  |  |  |
| **Pascoal et al. (2019)** |  |  |  |  |  |  |  |  |
| **Pieta et al. (2021)** |  |  |  |  |  |  |  |  |
| **Ruggieri et al. (1979)** |  |  |  |  |  |  |  |  |
| **Skouteris et al. (2005)** |  |  |  |  |  |  |  |  |

Additional file 6: Meta-analysis without outliers

These figures show the meta-analysis conducted excluding the 2 outlying studies that had very high effect sizes.


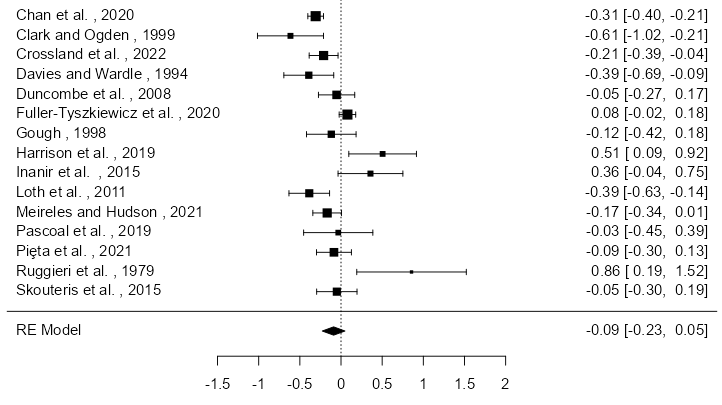


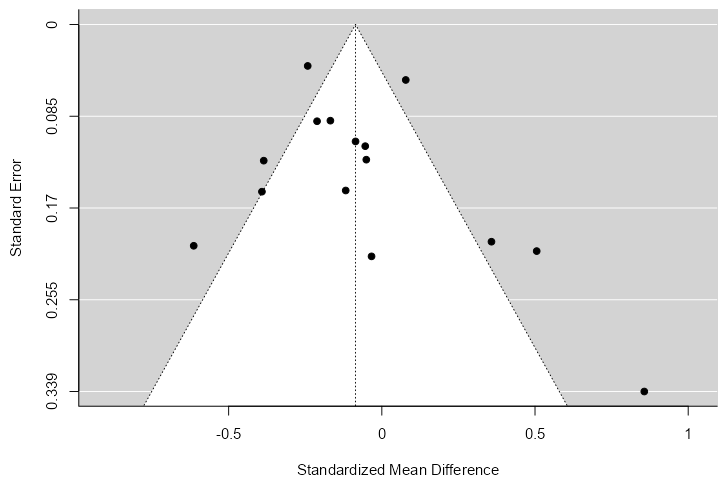

Supplement: Supplementary file 1 — Additional file 1. [file 12884_2023_5930_MOESM1_ESM.docx]
